# The Elder Justice Act and the Pandemic

**DOI:** 10.1093/geroni/igab046.1500

**Published:** 2021-12-17

**Authors:** Robert Blancato

**Affiliations:** Elder Justice Coalition, Washington, District of Columbia, United States

## Abstract

This session will provide updates on how the pandemic led to horrific situations in long-term care facilities and how the pandemic influenced major federal efforts to address elder abuse, neglect, and exploitation.

